# The Effect of Neutral Peritoneal Dialysis Solution with Low Glucose-Degradation-Product on the Fluid Status and Body Composition – A Randomized Control Trial

**DOI:** 10.1371/journal.pone.0141425

**Published:** 2015-10-28

**Authors:** Cheuk-Chun Szeto, Bonnie C. H. Kwan, Kai-Ming Chow, Phyllis M. S. Cheng, Vickie W. K. Kwong, Agnes S. M. Choy, Man-Ching Law, Chi-Bon Leung, Philip K. T. Li

**Affiliations:** Carol & Richard Yu Peritoneal Dialysis Research Centre, Department of Medicine & Therapeutics, Prince of Wales Hospital, The Chinese University of Hong Kong, Shatin, Hong Kong, China; Medical University of Graz, AUSTRIA

## Abstract

**Background:**

Previous studies report conflicting results on the benefit of peritoneal dialysis (PD) patients treated with low glucose degradation product (GDP) solution. The effects of low GDP solution on body fluid status and arterial pulse wave velocity (PWV) have not been studied.

**Methods:**

We randomly assigned 68 incident PD patients to low GDP (Intervention Group) or conventional solutions (Control Group); 4 dropped off before they received the assigned treatment. Patients were followed for 52 weeks for changes in ultrafiltration, residual renal function, body fluid status and arterial PWV.

**Result:**

After 52 weeks, Intervention Group had higher overhydration (3.1 ± 2.6 vs 1.9 ± 2.2 L, p = 0.045) and extracellular water volume (17.7 ± 3.9 vs 15.8 ± 3.1 L, p = 0.034) than Control Group. There was no significant difference in PWV between groups. There was no significant difference in residual renal function between the Groups. Intervention Group had lower ultrafiltration volume than Control Group at 4 weeks (0.45 ± .0.61 vs 0.90 ± 0.79 L/day, p = 0.013), but the difference became insignificant at later time points. Intervention Group had lower serum CRP levels than Control Group (4.17 ± 0.77 vs 4.91 ± 0.95 mg/dL, p < 0.0001).

**Conclusion:**

Incident PD patients treated with low GDP solution have less severe systemic inflammation but trends of less ultrafiltration, and more fluid accumulation. However, the effects on ultrafiltration and fluid accumulation disappear with time. The long term effect of low GDP solution requires further study.

**Trial Registration:**

ClinicalTrials.gov NCT00966615

## Introduction

Long term peritoneal dialysis (PD) by bio-incompatible solution has been proposed to be the cause of progressive loss of peritoneal permeability [1,2]. Amongst the ingredients in conventional PD solution, its acidic pH and the presence of glucose-degradation-product (GDP) are probably the major factors resulting in bio-incompatibility [3,4]. In recent years, a double-chamber bag Stay-Safe^®^ Balance system (Fresenius Medical Care, Bad Homburg, Germany) was developed. The ready-to-use solution has a physiological pH and a highly reduced amount of GDP [5].

A number of early studies suggested beneficial effects of the lactate-based pH-neutral solution on several components of the peritoneum [6–8]. The clinical benefit of this neutral low GDP solution, however, remains unclear. In a European multicenter prospective crossover trial that compared conventional solution with the new neutral solution [9], patients treated with the new solution had an improved profile of dialysate mesothelial markers. In our previous study, the use of neutral pH, low GDP solution resulted in a superior profile of PD effluent mesothelial cell marker and a lower degree of systemic inflammation as compared to conventional PD solution [10]. Other studies have reported variable and sometimes conflicting effects of low GDP solutions on ultrafiltration volume, urine output, decline of residual renal function, and peritonitis rate [9–17]. More importantly, there are no published data on the effect of low GDP solution on the overall body fluid status or arterial stiffness of PD patients. In the present study, we compare a double-chamber bag Stay-Safe^®^ Balance system and the conventional glucose-based solution in terms of nutritional status, arterial stiffness, and body composition and fluid status.

## Patients and Methods

The study was approved by our local clinical research ethics committee (Joint Chinese University of Hong Kong-New Territories East Cluster Clinical Research Ethics Committee). The study procedure was performed according to the Declaration of Helsinki and registered at ClinicalTrials.gov (ID NCT00966615).

### Overall arrangement

We recruited new adult continuous ambulatory peritoneal dialysis (CAPD) patients from February 2011 to July 2013. After written informed consent, they were randomized by a standard randomization table, which was kept by a third party, to receive the Balance System (Intervention Group) or a disconnect system with glucose-based dialysis solution (Stay-Safe^®^, Fresenius Medical Care, Germany) (Control Group). The biochemical composition of the two solutions is summarized in S1 Table. We excluded patients who were unlikely to survive, planned to have elective living-related kidney transplant, or transfer to other renal center within 6 months. Training for CAPD exchange was performed according to our routine clinical practice. All patients received home-based CAPD treatment after training was completed. The use of Stay-Safe^®^ solution has been the usual care of around half of our PD population in the past 10 years.

### Clinical follow up

Patients were followed at 0, 4, 8, 16, 24, 32, 40 and 52 weeks. Except for the dialysis solution preparation, the clinical management was identical for the two groups. During each follow up visit, we measured body weight, blood pressure, presence of edema, and compliance to dialysis exchange by direct questioning. Hemoglobin level, serum electrolytes, urea and creatinine were checked upon each clinic visit. Dialysis regimen was adjusted according to clinical assessment of fluid status, urine output, and record of ultrafiltration by the patient. A glucose polymer solution was not used because of the difference in connection tubing. Physicians were blinded from the result of bioimpedance spectroscopy or arterial pulse wave velocity throughout the study. The use of anti-hypertensive drug was quantified by the numbers and defined daily doses of antihypertensive drugs (one defined daily dose is the average maintenance dose per day for adults) [18]. Hypertonic cycle was defined as any PD exchange with dextrose concentration higher than 1.5%, which was generally used when the patient had inadequate ultrafiltration or clinical evidence of fluid overload.

### Bioimpedance Spectroscopy

Body composition was assessed at 4, 24 and 52 weeks by bioimpedance spectroscopy (Body Composition Monitor, Fresenius Medical Care, Germany) with PD solution instilled. The method of bioimpedance spectroscopy has been described previously [19]. We computed the following parameters from this test: total body water, intracellular water, and extracellular water, lean tissue mass (LTM), adipose tissue mass (ATM), and volume of over hydration.

### Arterial Pulse Wave Velocity Study

Pulse wave velocity (PWV), an index of aortic stiffness, was measured at 4 and 52 weeks using an automatic computerized recorder and the results are analyzed using the Complior^®^ SP program (Artech Medical, France). The method of PWV measurement has been described previously [20].

### Assessment of peritoneal transport

Peritoneal transport was assessed at 4 and 52 weeks. We used the standard PET as described by Twardowski [21]. Dialysate-to-plasma ratios of creatinine (D/P) at 4 hours was calculated after correction of glucose interference [22]. Mass transfer area coefficients of creatinine (MTAC) normalized for body surface area (BSA) was calculated by the formula described by Krediet [23].

### Dialysis adequacy and nutritional status

Dialysis adequacy was assessed at 4, 24 and 52 weeks by 24-hour dialysate and urine collections. Total Kt/V, a measurement of body urea clearance provided by dialysis and the residual kidney function, was determined by standard methods. Residual glomerular filtration rate (GFR) was calculated as the average of 24-hour urinary urea and creatinine clearance [24]. We also computed the normalized protein nitrogen appearance (NPNA) by the Bergstrom’s formula [25], and fat-free edema-free body mass (FEBM) by creatinine kinetics according to the formula of Forbes and Bruining [26].

In addition to FEBM and NPNA, nutritional status was represented by Subjective global assessment (SGA) score [27], the comprehensive malnutrition-inflammation score (MIS) [28], serum albumin and C-reactive protein (CRP) at 4, 24 and 52 weeks. SGA was measured in a 7-point scale; a higher score means better nutrition [27]. MIS a combination score of 10 items, with a total score of 30; a higher score means worse nutrition [28]. Serum C-reactive protein (CRP) was measured by the Tina-quant CRP (Latex) ultra-sensitive assay (Roche Diagnostics GmbH, Mannheim, Germany).

### Clinical Outcome

All patients were followed for 12 months. The primary outcome measures are the change in body composition and arterial pulse wave velocity. The latter was chosen as a primary outcome measure because our previous study showed that asymptomatic fluid overload in PD patients may result in worsening of arterial PWV [19]. Secondary outcomes include nutritional and adequacy indices, peritoneal transport characteristics, residual renal function, peritonitis-free survival, hospitalization, and patient survival and technique survival. Technique failure is defined as transfer to other modes of renal replacement therapy.

### Sample size

The sample size was estimated by the Power Analysis and Sample Size for Windows software (PASS 2000, NCSS, Kaysville, Utah), calculated on the base of simple comparison. Based on our previous studies [20,29], aortic PWV is expected to be 10.2 ± 1.6 m/sec. We assumed a difference of 1 m/sec in the PWV to be clinically meaningful. Group sizes of 45 achieve 80% power to detect such a difference of PWV, with a significance level (alpha) of 0.05. Allowing for 10% drop out rate, the study was estimated to require 100 patients in total.

### Statistical Analysis

Statistical analysis is performed by SPSS for Windows software version 18.0 (SPSS Inc., Chicago, IL). All data are expressed in mean ± standard deviation unless otherwise specified. Since all the primary outcome measures were serial biochemical and laboratory parameters, which were not available for patients who did not receive the assigned treatment, only the result of per protocol analysis is presented. Parameters between groups are compared by Chi-square test, Student’s t test, or Mann-Whitney U test as appropriate. Serial data are compared by paired Student’s t test. Multivariate analysis was not performed to adjust for confounding factors because of the small number of patients. Peritonitis-free survival between groups were calculated by Kaplan Meier survival plot and compared by the log rank test. A P value of less than 0.05 is considered statistically significant; Bonferroni method is used to correct for multiple comparisons. All probabilities are two-tailed.

## Results

**Fig 1:**
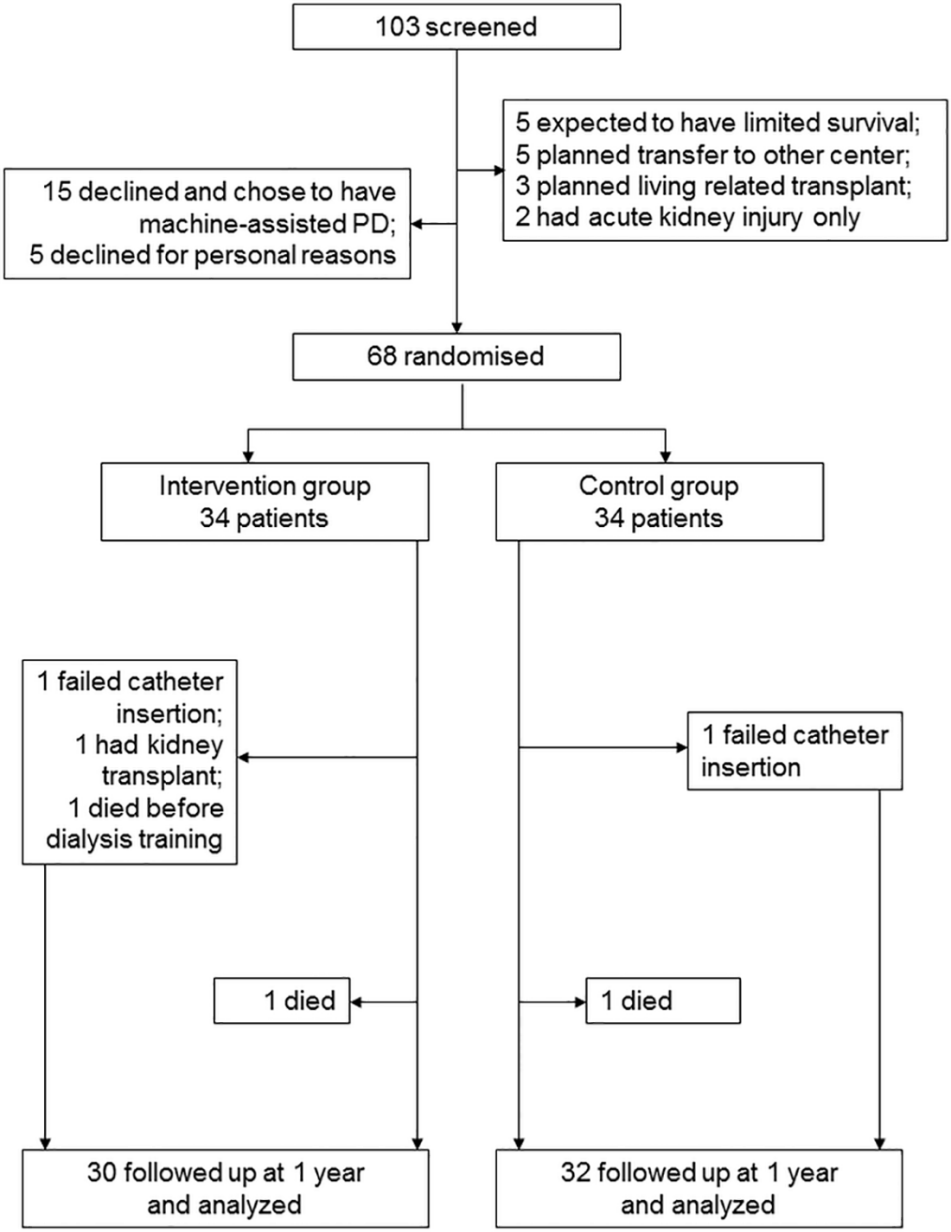
Consort diagram of the trial profile.

During the recruitment period, we obtained consent from 68 patients, but 4 were excluded before treatment was started. Fig 1 depicts the Consort flow diagram that summarizes the trial profile. The baseline clinical characteristics of the 64 patients who received their assigned treatment are summarized in Table 1. In essence, the Intervention Group had more diabetic patients (64.5% vs 33.3%), tend to be older (62.9 vs 57.7 years), and had higher Charlson’s scores than the Control Group.

**Table 1:** Baseline characteristics of the patients.

	Intervention Group	Control Group
No. of patient	31	33
Sex (M:F)	17:14	13:20
Age (years)	62.9 ± 12.1	57.7 ± 9.9
Body height (cm)	162.4 ± 8.8	160.9 ± 8.3
Body mass index (kg/m^2^)	24.4 ± 3.1	23.0 ± 3.1
Diagnosis, no. of cases (%)		
Glomerulonephritis	6 (19.4%)	12 (36.4%)
Diabetic nephropathy	18 (58.1%)	9 (27.3%)
Hypertensive nephrosclerosis	6 (19.4%)	4 (12.1%)
Polycystic kidney	0	1 (3.0%)
Obstruction	1 (3.2%)	4 (12.1%)
Others / unknown	0	3 (9.1%)
Major comorbidity, no. of cases (%)		
Diabetes	20 (64.5%)	11 (33.3%)
Coronary heart disease	4 (12.9%)	4 (12.1%)
Cerebrovascular disease	7 (22.6%)	5 (15.2%)
Charlson’s Index score	6.2 ± 2.1	5.1 ± 2.2

Data are presented as mean ± standard deviation.

### Body composition and fluid status

Blood pressure, dialysis regimen, use of diuretic agents, and peritoneal transport characteristics are compared in Table 2. Both at 4 and 52 weeks, the Intervention Group had a trend of using more hypertonic cycle and diuretic agents than the Control Group, although none of the difference was significant. The total glucose load during the study period was marginally higher in the Intervention than Control Group (38.8 ± 12.2 vs 33.8 ± 7.5 kg, p = 0.06). At 4 weeks, the Intervention Group had higher D/P creatinine at 4 hours and MTAC creatinine than the Control Group (Table 2), but the difference became insignificant by 52 weeks.

**Table 2:** Blood pressure, lipid profile, dialysis regimen, use of diuretic agents, peritoneal transport characteristics, fluid status, and arterial pulse wave velocity during the study period.

	4 weeks			52 weeks		
	Intervention Group	Control Group	P value	Intervention Group	Control Group	P value
Blood pressure						
systolic (mmHg)	135.7 ± 18.5	134.8 ± 28.2	p = 0.9	140.5 ± 28.1	139.7 ± 24.5	p = 0.9
diastolic (mmHg)	70.5 ± 13.8	73.1 ± 17.5	p = 0.5	78.0 ± 15.4	72.6 ± 12.0	p = 0.13
no. of antihypertensive drugs	1.9 ± 0.7	1.8 ± 0.7	p = 0.6	2.1 ± 0.7	1.8 ± 1.0	p = 0.2
defined daily dose of antihypertensive drugs	2.5 ± 1.1	2.3 ± 1.2	p = 0.6	2.5 ± 1.0	2.2 ± 1.4	p = 0.4
Lipid profile (mmol/l)						
total cholesterol	5.06 ± 1.60	4.68 ± 0.94	p = 0.3	5.25 ± 1.63	5.21 ± 1.31	p = 0.9
triglyceride	1.94 ± 1.16	1.72 ± 1.02	p = 0.4	2.27 ± 1.53	1.58 ± 0.89	p = 0.036
LDL cholesterol	2.87 ± 1.30	2.55 ± 0.78	p = 0.2	2.98 ± 1.34	3.06 ± 1.06	p = 0.8
HDL cholesterol	1.23 ± 0.43	1.36 ± 0.45	p = 0.2	1.28 ± 0.51	1.43 ± 0.50	p = 0.3
Dialysis regimen						
exchange volume (6:8 L/day), no. of patients	31:0	33:0		27:3	31:1	p = 0.3
hypertonic cycle, no. of patients	9	5	p = 0.2	14	8	p = 0.08
Glucose load (g/day)	101.5 ± 37.1	89.1 ± 19.4	p = 0.1	111.6 ± 38.2	96.1 ± 28.0	p = 0.07
Diuretic usage						
frusemide (mg/day)*	80 (20–205)	40 (0–250)	p = 0.3	40 (0–250)	40 (0–250)	p = 0.7
other diuretics, no. of patients	5	2	p = 0.2	5	1	p = 0.1
Peritoneal transport						
D/P creatinine at 4 hour	0.743 ± 0.101	0.667 ± 0.124	p = 0.01	0.702 ± 0.082	0.651 ± 0.108	p = 0.07
MTAC creatinine	13.0 ± 5.3	10.3 ± 4.4	p = 0.03	10.8 ± 3.5	10.1 ± 4.9	p = 0.6
Overhydration (L)						
diabetic patients	3.89 ± 3.02	3.45 ± 2.52	p = 0.7	3.73 ± 2.96	2.67 ± 2.19	p = 0.3
non-diabetic patients	1.25 ± 0.96	1.80 ± 1.82	p = 0.4	1.96 ± 1.27	1.48 ± 2.07	p = 0.5
E:I fluid volume ratio						
diabetic patients	1.02 ± 0.15	1.02 ± 0.17	p = 0.9	1.02 ± 0.16	0.98 ± 0.15	p = 0.5
non-diabetic patients	0.85 ± 0.09	0.88 ± 0.11	p = 0.6	0.93 ± 0.13	0.86 ± 0.14	p = 0.2
Carotid-femoral PWV (m/sec)						
diabetic patients	13.03 ± 2.70	13.00 ± 2.30	p = 0.9	14.41 ± 3.01	13.21 ± 1.26	p = 0.2
non-diabetic patients	10.29 ± 1.88	10.52 ± 2.07	p = 0.8	10.90 ± 1.84	11.08 ± 1.97	p = 0.8

LDL, low density lipoprotein; HDL, high density lipoprotein; D/P, dialysate-to-plasma concentration ratio; MTAC, mass transfer area coefficient; E:I, extracellular-to-intracellular; PWV, pulse wave velocity. Data described in mean ± standard deviation or *median (inter-quartile range).

**Fig 2:**
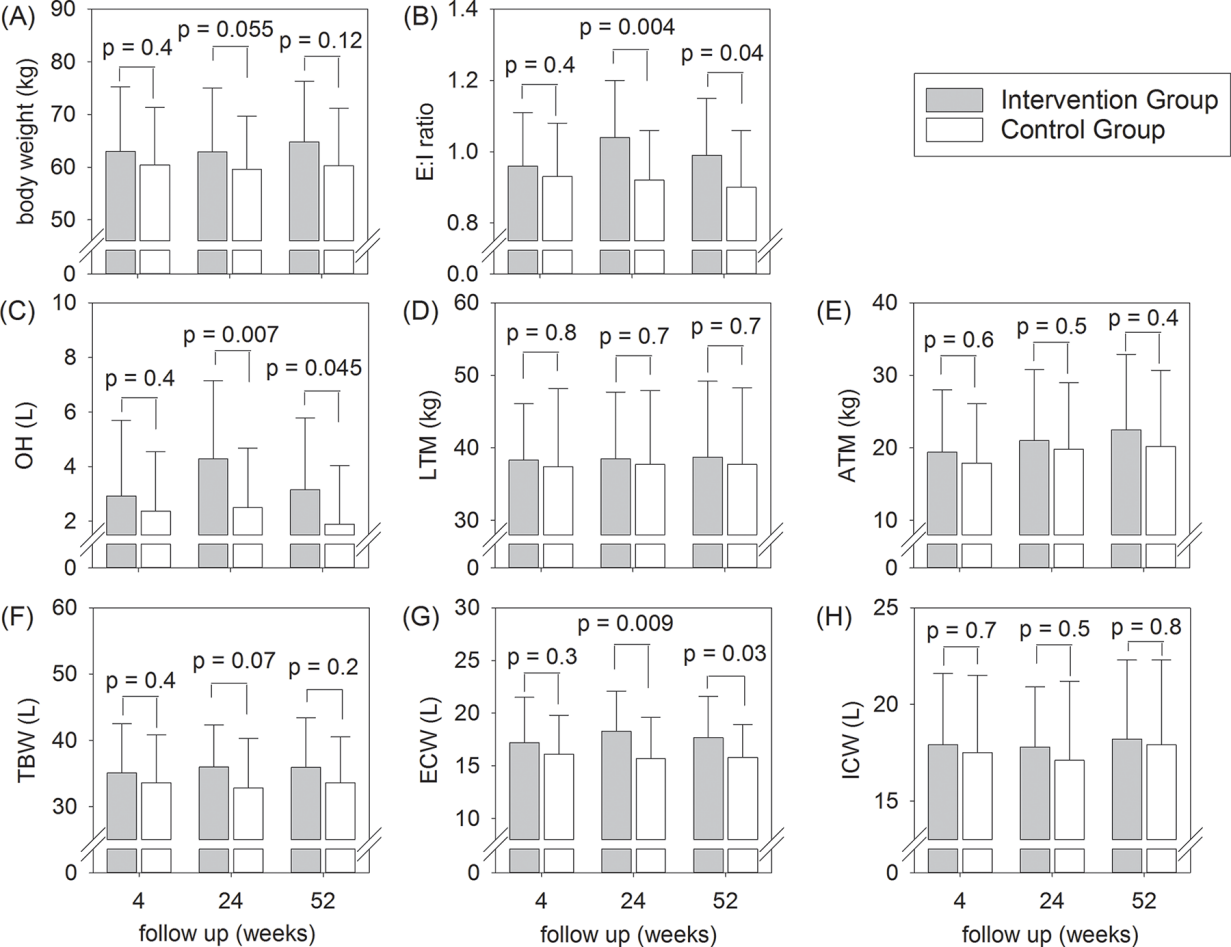
Body composition and fluid status during the study period. (A) body weight; (B) extracellular-to-intracellular fluid (E:I) ratio; (C) overhydration (OH); (D) lean tissue mass (LTM); (E) adipose tissue mass (ATM); (F) total body water (TBW); (G) extracellular water (ECW); and (H) intracellular water (ICW). Error bars denote standard deviations; P values denote the comparison between the Intervention and Control Groups by the unpaired Student’s t test.

The body composition and fluid status of the two groups are compared in Fig 2. In essence, there was no difference in the baseline body composition and fluid status between the two groups. After 24 weeks, the Intervention Group had higher overhydration (4.3 ± 2.9 vs 2.5 ± 2.2 L, p = 0.007), extracellular water volume (18.3 ± 3.8 vs 15.7 ± 3.9 L, p = 0.009), E:I ratio (1.04 ± 0.16 vs 0.92 ± 0.14, p = 0.004), total body water (36.0 ± 6.3 vs 32.8 ± 7.5 L, p = 0.07), and body weight (62.9 ± 12.1 vs 59.6 ± 10.1 kg, p = 0.055), although the results of total body water and body weight are not significant. These differences persisted but became less marked by 52 weeks. When diabetic and non-diabetic patients were separately analyzed, there was no difference in overhydration or E:I ratio between Intervention and Control Group at any time point (Table 2). There was no significant difference in lean tissue mass (LTM) or adipose tissue mass (ATM) between the two Groups throughout the study period. ATM increased modestly in both group, while LTM remained static, during follow up.

### Arterial pulse wave velocity

**Fig 3:**
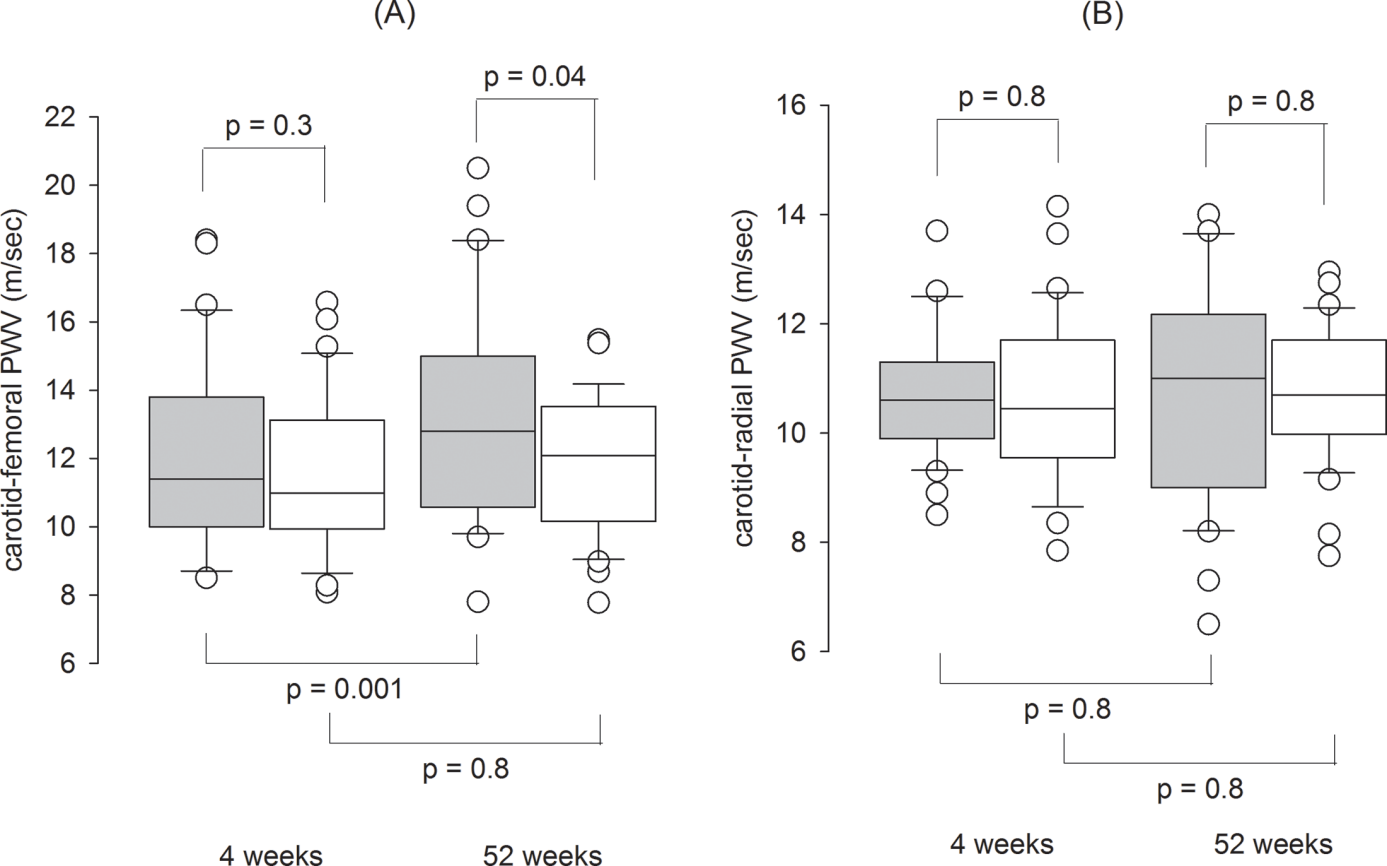
Change in pulse wave velocity (PWV) during the study period. (A) carotid-femoral; and (B) carotid-radial PWV. Whister-box plots, with boxes indicate median, 25th and 75th percentiles, whiskers indicate 5th and 95th percentiles. P values depict the comparison between the Intervention and Control Groups by the unpaired Student’s t test. (Grey box, Intervention Group; White box, Control Group).

The arterial pulse wave velocity of the two groups are compared and summarized in Fig 3. Both carotid-femoral and carotid-radial PWV were similar between the Intervention and Control Groups at 4 weeks. After 52 weeks of PD, carotid-femoral PWV had a modest but significant increase in the Intervention Group (12.1 ± 2.8 to 13.2 ± 3.1 m/sec, paired t-test, p = 0.001), while it remained static in the Control Group. Carotid-radial PWV remained static in both Groups. At 52 weeks, the Intervention Group had slightly higher carotid-femoral PWV than the Control Group (13.2 ± 3.1 vs 11.8 ± 2.0 m/sec, p = 0.04), but the carotid-radial PWV were similar. When diabetic and non-diabetic patients were separately analyzed, there was no difference in PWV between the two groups at any time point (Table 2).

### Residual renal function, inflammation, and nutritional status

**Fig 4:**
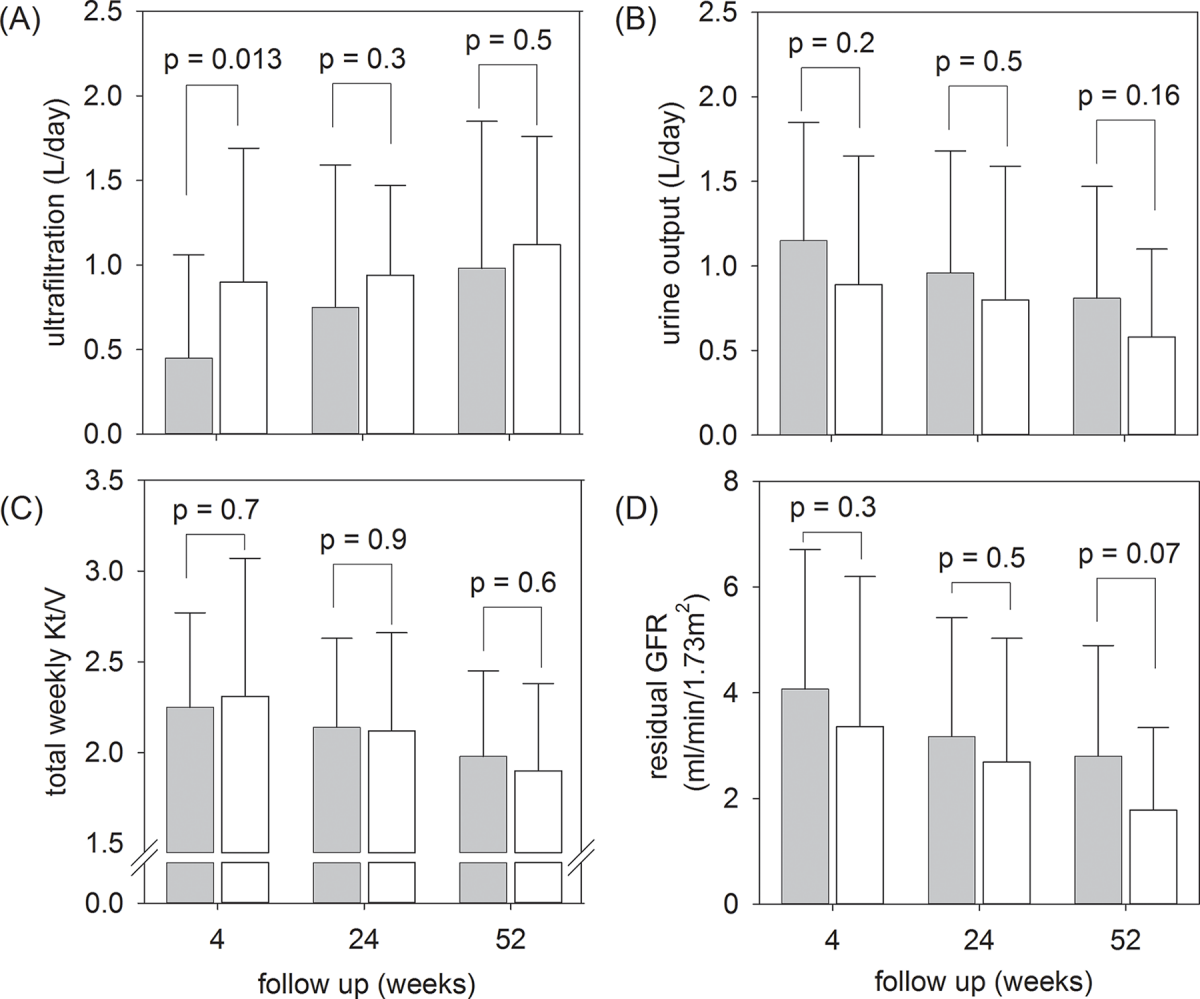
Fluid removal, dialysis adequacy, and residual renal function during the study period. (A) ultrafiltration volume by peritoneal dialysis; (B) urine output; (C) weekly total Kt/V; and (D) residual glomerular filtration rate (GFR). Error bars denote standard deviations; P values denote the comparison between the Intervention and Control Groups by Mann-Whitney U test. (Grey box, Intervention Group; White box, Control Group).

Fluid removal, dialysis adequacy, and residual renal function of the two groups during the study period are compared in Fig 4. By univariate analysis, the Intervention Group had a lower ultrafiltration volume than the Control Group 4 weeks after PD (0.45 ± .0.61 vs 0.90 ± 0.79 L/day, p = 0.013), but the difference became insignificant at later time points. In contrast, there was a progressive decline in urine output, residual GFR, and total Kt/V, together with a corresponding increase in ultrafiltration volume in both Groups during the study period. At 52 weeks, the Intervention Group had a trend of higher urine output (0.81 ± 0.66 vs 0.58 ± 0.52 L/day, p = 0.16) and residual GFR (2.80 ± 2.09 vs 1.78 ± 1.56 ml/min/1.73m^2^, p = 0.07) than the Control Group, but the difference was not significant. After one year, 5 patients from each group progressed to anuria.

**Fig 5:**
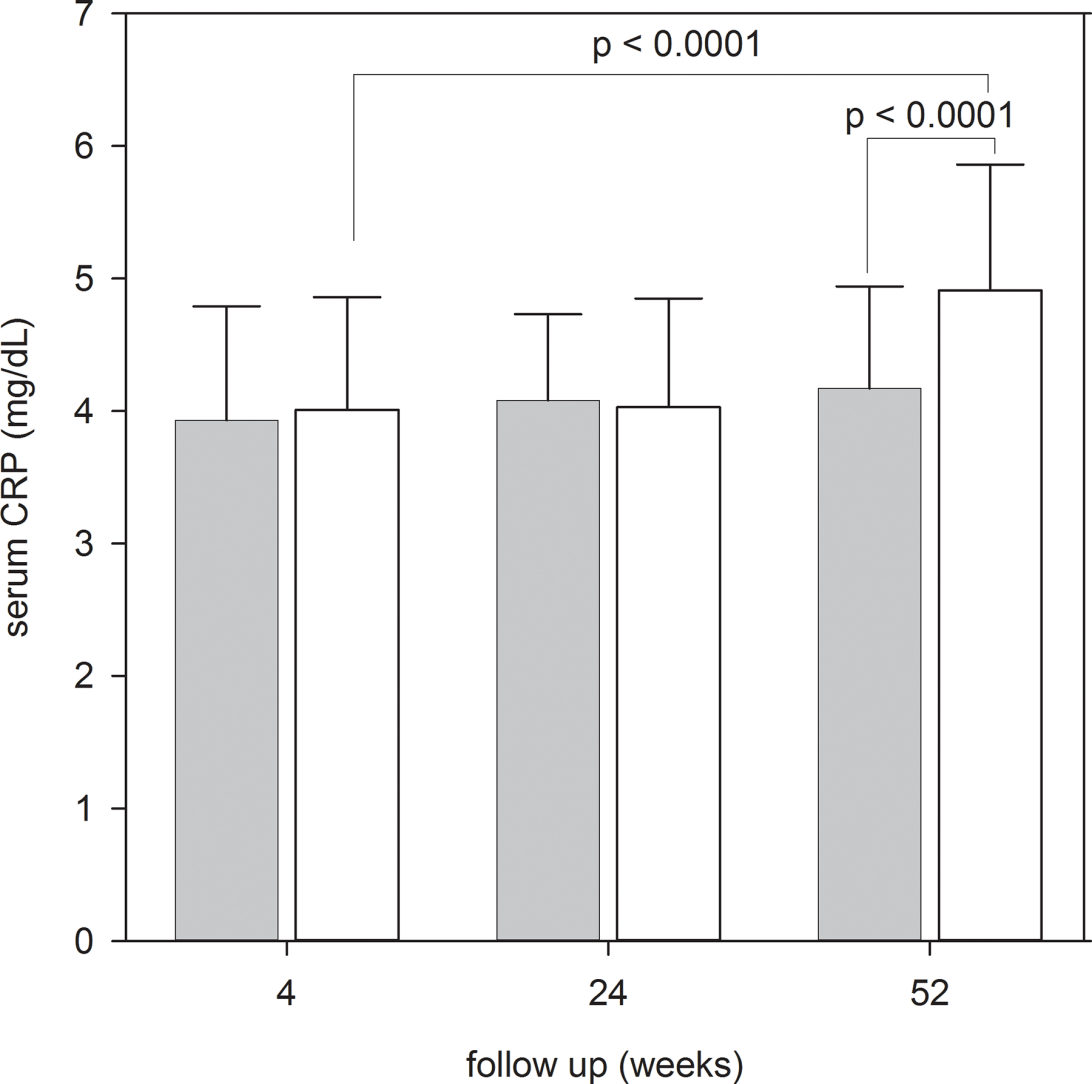
Serum C-reactive protein (CRP) during the study period. Error bars denote standard deviations; P values denote the comparison between the Intervention and Control Groups by the Student’s t test. (Grey box, Intervention Group; White box, Control Group).

During the first year of PD, the Control Group had a significant increase in serum CRP level, while the Intervention Group also had an insignificant trend of rising CRP (Fig 5). At 52 weeks, the Intervention Group had a significantly lower serum CRP level than the Control Group (4.17 ± 0.77 vs 4.91 ± 0.95 mg/dL, p < 0.0001). There was also a trend of progressive decline in SGA score and a corresponding increase in MIS in both Groups during the study (Table 3), but the change was not statistically significant. There was no significant difference in other nutritional indices between the two Groups (Table 3).

**Table 3:** Nutritional indices during the study period.

	Intervention Group			Control Group		
	4 weeks	24 weeks	52 weeks	4 weeks	24 weeks	52 weeks
Hemoglobin (g/dL)	9.54 ± 1.77	9.87 ± 1.47	9.66 ± 1.65	9.45 ± 1.13	9.42 ± 1.42	9.37 ± 1.34
Serum albumin (g/L)	33.9 ± 4.3	34.7 ± 3.8	35.2 ± 3.1	35.0 ± 4.2	35.3 ± 3.1	35.3 ± 3.1
NPNA (g/kg/day)	1.08 ± 0.21	1.18 ± 0.23	1.15 ± 0.17	1.19 ± 0.22	1.20 ± 0.26	1.15 ± 0.18
FEBM (%)	38.5 ± 8.3	43.6 ± 10.8	46.7 ± 10.7	45.0 ± 11.4	48.8 ± 17.0	51.7 ± 12.9
MIS	8.0 ± 3.4	10.1 ± 4.2	11.1 ± 3.5	9.8 ± 4.4	9.0 ± 3.8	10.9 ± 4.0
SGA score	5.5 ± 0.8	5.1 ± 0.9	4.9 ± 0.8	5.2 ± 1.0	5.2 ± 0.9	5.1 ± 0.9

NPNA, normalized protein nitrogen appearance; FEBM, fat-free edema-free body mass; MIS, malnutrition inflammation score; SGA, subjective global assessment. Data are presented as mean ± standard deviation.

### Peritonitis, hospitalization, survival, and adverse reactions

Peritonitis and hospital admission of the two groups during the study period are compared in Table 4. The peritonitis-free survival at 52 weeks was 74.2% and 75.8% for in the Intervention and Control Groups, respectively (p = 0.9) (S1 Fig). During the study period, one patient from each Group died, and none had kidney transplant after CAPD was started or transfer to long-term hemodialysis. Analysis of patient or technique survival is not performed due to the small number of event. There was no adverse reactions reported in either study group.

**Table 4:** Peritonitis and hospital admission during the study period.

	Intervention Group	Control Group	P value
No. of patient	31	33	
Peritonitis			
no. of peritonitis episodes	9	11	p = 0.8
no. of patients being peritonitis-free	23	25	p = 0.9
Hospital admission			
all cause			
no. of hospital admission	53	35	p = 0.4
duration of hospitalization (days)	315	249	p = 0.3
admission for CVD			
no. of hospital admission	14	7	p = 0.4
duration of hospitalization (days)	80	78	p = 0.4
Death, no. of patient (cause)	1 (CVD)	1 (infection)	

CVD, cardiovascular disease.

## Discussion

In this randomized control study, we found that new PD patients treated with low GDP solution had more fluid accumulation, less ultrafiltration from PD, but more urine output than patients treated with conventional PD solutions. As compared to the control group, patients receiving the low GDP solution also had less severe systemic inflammation but higher carotid-femoral arterial PWV after one year of PD.

Although there are a number of randomized control trials on the possible benefit low GDP solutions [30], our present study is the first one that examines body fluid status in detail. Three previous studies reported no significant difference in body weight between patients treated with low GDP solutions and those with conventional ones at 12 and 24 months [12–14]. Our result indicates that after 6 months of PD, although low GDP solution has no substantial effect on the overall body weight or blood pressure, these patients tend to have more overhydration, extra-cellular water (ECW) volume, and extracellular-to-intracellular fluid (E:I) ratio than patients treated with conventional solutions. However, it is important to note the effect of low GDP solution on overhydration and ECW volume became less marked at 12 months, probably because of the loss of residual renal function in the Control Group.

The cause of excessive fluid accumulation in the Intervention Group is unclear. In spite of randomization, there were more diabetic patients in the Intervention Group, which may potentially affect the result. A previous report shows that diabetic patients have substantial alterations of the peritoneal structure [31], which may explain the higher D/P creatinine, worse ultrafiltration, and more fluid overload in our Intervention Group. There were also slightly more men in the Intervention Group, which might also contribute to more fluid overload in this group. In the present study, we did not document the dietary intake, and we cannot exclude the possibility that the Intervention Group had a better appetite and more salt and water intake than the Control Group. On the other hand, the excessive fluid accumulation could be the result of less fluid removal by ultrafiltration (see Fig 4). Several previous studies found a lower ultrafiltration volume with the use of low GDP solutions [9,14,15], although others showed no significant effect [10,13,16]. In the balANZ Trial, patients in the biocompatible group had significantly lower ultrafiltration at 3 and 6 months but not later time points [12,32]. Taken together, available data suggest a modest negative effect of low GDP solution on peritoneal ultrafiltration. However, the effect seems to disappear with time, and the clinical relevance is uncertain.

We found no difference in residual GFR decline, urine output, or progression to anuria between the two groups. Although several studies observed better preservation of residual renal function by low GDP solutions [14–16], three major randomized control trials found that low GDP solution has no effect in slowing the rate of GFR decline [12,13,17]. In our study, the use of diuretic agents was similar between the Groups, suggesting that the higher urine output amongst patients treated with low GDP solution is not related to diuretic therapy.

We found conflicting changes in arterial PWV and serum CRP levels. After 12 months of PD, patients treated with low GDP solution had more marked increase in carotid-femoral PWV, indicating progressive arterial stiffening, but a lower serum CRP level, suggesting an amelioration of the systemic inflammatory state. Notably, the effect of low GDP solution on arterial stiffness has not been examined in previous studies. The rapid progression of arterial stiffness in the Intervention Group is probably explained by the imbalance in baseline characteristics, because the difference disappears after adjusting for confounding factors. However, the progression of arterial stiffness may partly represent the chronic effect of overhydration, supporting the notion that long-standing mild fluid overload has clinical consequence, which has been reported by several groups previously [19,33]. On the other hand, the effect of low GDP solution on systemic inflammation remains uncertain. While two previous studies showed that the use of low GDP solution resulted in lower serum CRP levels [10,17], others found no difference [13,14,16].

Although our results do not suggest a favorable effect of low GDP solution on body fluid status or arterial pulse wave velocity, there could have been bias in our study because, despite randomization, the proportion of diabetic patient is significantly higher in the Intervention Group and this group of patients tend to be older. Although the result remains similar when diabetic and non-diabetic patients are analyzed separately, the number of patient is small with subgroup analysis and there could be statistical error.

There are other limitations of our study. First, the sample size is small and we do not have sufficient power to determine a small but clinically important difference in residual renal function, peritonitis or hospitalization rate. We encountered unexpected difficulties during recruitment, and we could only recruit 68 out of 100 patients as originally planned. However, our sample size was estimated by the difference in PWV, of which we do find a difference between the Groups. Furthermore, the duration of our study may not be sufficient. As noticed in our study as well as the balANZ trial [12], the initial undesirable effects of low GDP solution on ultrafiltration, peritoneal transport, and body fluid status diminish with time. It remains unknown whether prolonged use of low GDP solution would help to preserve peritoneal function. Long term study is necessary to test this hypothesis.

Secondly, we used multiple-frequency bioimpedance spectroscopy for the measurement of body composition and fluid status. Although the method has a good overall agreement to the gold-standard isotope dilution techniques, the intra-individual variability is considerable [34]. Unfortunately, we do not have baseline fluid status before dialysis was started, or data on cardiac function. Similarly, we do not have data on peritoneal transport before dialysis because peritoneal transport characteristics change significantly within the first month of PD [35] and determination of “baseline” peritoneal transport status was not possible.

In summary, incident PD patients treated with low GDP solution have less severe systemic inflammation but trends of less ultrafiltration, and more fluid accumulation. However, the effects on ultrafiltration and fluid accumulation disappear with time. The long term effect of low GDP solution requires further study.

## Supporting Information

S1 CONSORT Checklist CONSORT checklist. (DOC)

S1 Fig Kaplan-Meier plot of peritonitis-free survival. (TIF)

S1 File Participant-level data. (XLSX)

S1 Protocol Trial protocol. (DOC)

S1 Table Biochemical composition of the peritoneal dialysis solutions. (DOCX)
