# Supplementary figures and images for: The Effect of Neutral Peritoneal Dialysis Solution with Low Glucose-Degradation-Product on the Fluid Status and Body Composition – A Randomized Control Trial
Source: PLoS One. 2015 Oct 28;10(10):e0141425. doi: 10.1371/journal.pone.0141425 (PMC4625015; doi:10.1371/journal.pone.0141425)

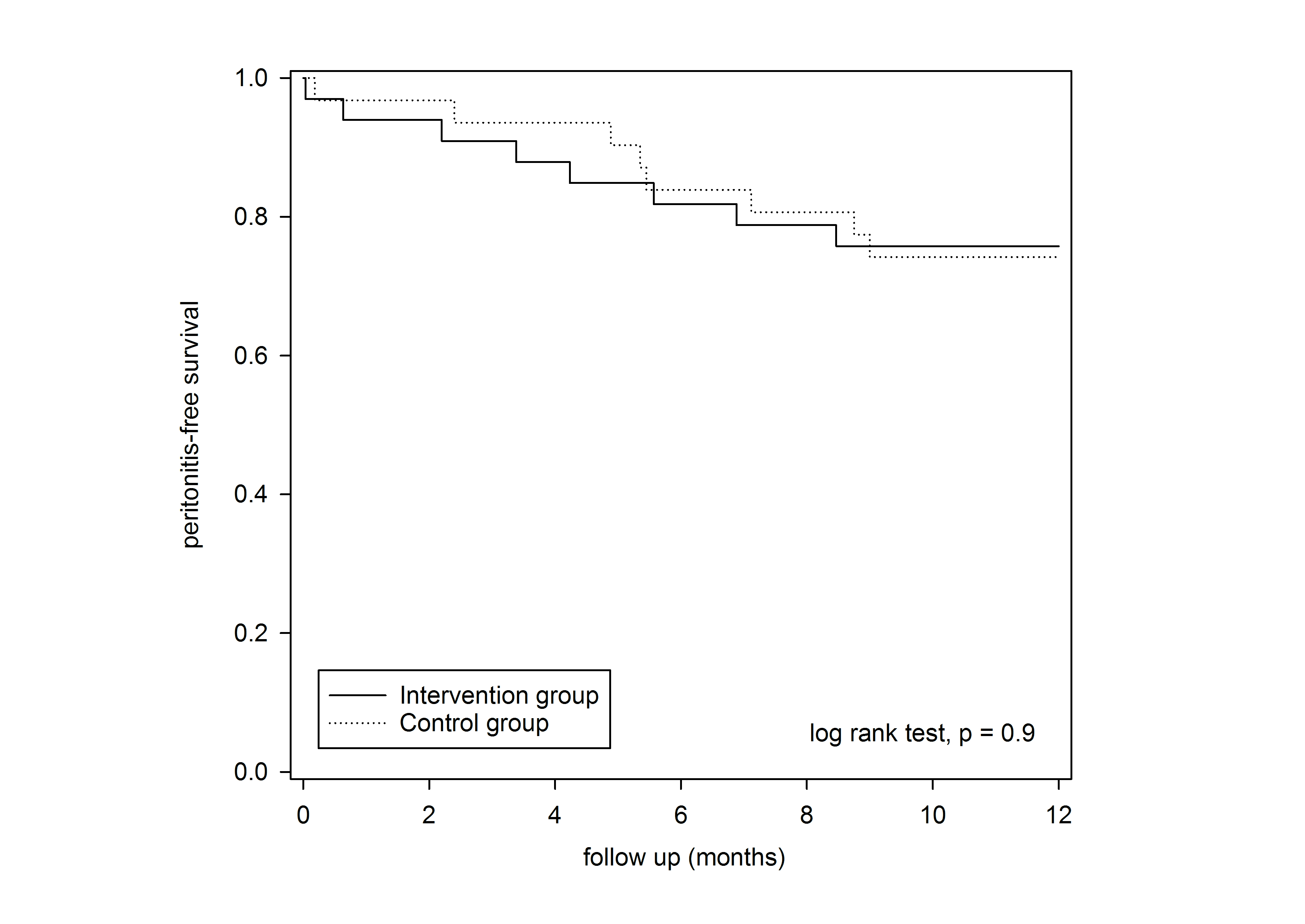

Supplement: S1 Fig — (TIF) [file pone.0141425.s002.TIF]
