# Supplementary material for: The Effect of Neutral Peritoneal Dialysis Solution with Low Glucose-Degradation-Product on the Fluid Status and Body Composition – A Randomized Control Trial
Source: PLoS One. 2015 Oct 28;10(10):e0141425. doi: 10.1371/journal.pone.0141425 (PMC4625015; doi:10.1371/journal.pone.0141425)
Supplement: S1 Protocol — (DOC) [file pone.0141425.s004.doc]

The Effect of Neutral Peritoneal Dialysis Solution With Minimal Glucose-Degradation-Product on the Fluid Status and Body Composition – A Randomized Control Trial

Principal Investigator

SZETO, Cheuk Chun, MD, FRCP, Professor

Co-Investigator

KWAN, Ching-Ha Bonnie, MBBS, MRCP(UK), Associate Professor

LI, Kam Tao Philip, MD, FRCP FACP, Consultant

Department of Medicine & Therapeutics, Prince of Wales Hospital, The Chinese University of Hong Kong, Shatin, Hong Kong, China.

ABSTRACT

Chronic utilization of bio-incompatible peritoneal dialysis (PD) solution has been implicated as a cause of progressive loss of peritoneal permeability and recurrent fluid overload in PD patients. Previous studies show that PD solution with neutral pH and low GDP resulted in a superior profile of PD effluent mesothelial cell marker and a lower degree of systemic inflammation as compared to conventional PD solution. We propose a prospective randomized control study to compare the arterial stiffness, nutrition and body fluid status between PD patients treated with conventional solution and those with neutral pH low GDP solution. We plan to study 100 new PD patients. They will be randomized to be treated with neutral pH low GDP solution or conventional solution. All patients will be followed for 52 weeks. In addition to routine clinical measurements, we will measure their body water composition by bioimpedance spectroscopic method, arterial pulse wave velocity by pressure transduction method, as well as radiographic parameters of intravascular volume status, based on the routine chest radiograph. Our study would help to define the clinical benefit of biocompatible PD solution.

BACKGROUND

Chronic utilization of bio-incompatible peritoneal dialysis (PD) solution has been implicated as a cause of progressive loss of peritoneal permeability [1,2]. Acidic pH and glucose-degradation-product (GDP) in the commercially available peritoneal dialysis solution are major contributing factors of bio-incompatibility [3,4]. Recently, a double-chamber bag Stay-Safe® Balance system (Fresenius Medical Care, Bad Homburg, Germany) became available. This system utilizes lactate-buffered peritoneal dialysis solution in a two-compartment bag offered in the Stay-Safe® disconnect system. The formation of GDP is greatly reduced by separating the glucose component of the solution (kept at very low pH) from the lactate component of the solution (kept at alkaline pH) during sterilization and storage. Immediately before infusion, the seam between the two chambers is opened, and the contents are mixed. The ready-to-use solution has a physiological pH in the range 6.8 to 7.4, and a highly reduced amount of a wide spectrum of GDP [5], including 3-deoxyglucosone, methylglyoxal, acetaldehyde and formaldehyde.

There is early evidence suggesting beneficial effects of the lactate-based pH-neutral solution on several components of the peritoneum. For example, exposure of human peritoneal mesothelial cells (HPMC) in vitro to conventional PD solution resulted in a significant reduction in IL-6 release, which was fully restored following exposure to Stay-Safe Balance solution [6]. While exposure to conventional PD solution resulted in a significant reduction in HPMC viability after just 3 to 5 days, no significant toxicity of the Stay-Safe Balance solution was observed for up to 13 days [6]. In addition, there was a better preservation of in vitro phagocyte function with the two-compartment, lactate-based neutral PD solution [7], and In vitro formation of advanced glycation end-product (AGE) was also substantially reduced [8].

There are, however, few clinical data on this lactate-based neutral PD solution. In a rat model of peritoneal dialysis, chronic exposure of the peritoneum to PD solution with low GDP and a physiologic pH reduced the intraperitoneal inflammatory reaction, peritoneal fibrosis [9], and peritoneal vasodilatation [10]. An observational study with 9 patients suggested that even short-term treatment (an average of 8 weeks) with the Stay-Safe Balance solution improved mesothelial cell mass as indicated by a rise in cancer antigen 125 (CA125) [6], although he peritoneal surface area is not measurably influenced [11]. In a European multicenter prospective crossover trial that compared conventional solution with the new neutral solution [12], patients treated with the new solution had an improved profile of dialysate mesothelial markers. In our recent randomized control study, we found that the use of neutral pH, low GDP solution resulted in a superior profile of PD effluent mesothelial cell marker and a lower degree of systemic inflammation as compared to conventional PD solution [13]. However, it remains unknown whether preservation of peritoneal function by low GDP solution could result in less systemic fluid overload, which is a major cardiovascular morbidity in PD patients [14].

OBJECTIVE

In a randomized control trial setting, we plan to study compare a double-chamber bag Stay-Safe® Balance system and the conventional glucose-based solution in terms of nutritional status, arterial stiffness, and body composition and fluid status.

PATIENTS AND METHODS

## Overall arrangement

We plan to recruit 100 new adult continuous ambulatory peritoneal dialysis (CAPD) patients: 50 with the Balance System (Balance Group) and 50 with disconnect system with glucose-based dialysis solution (Stay-Safe®, Fresenius Medical Care, Germany) (Control Group). We will exclude patients who are unlikely to survive, planned to have elective living-related kidney transplant, or transfer to other renal center within 6 months. Informed consent will be obtained at the time of Tenckhoff insertion. Individuals will be randomized by drawing sealed envelops, which are prepared and then maintained by a third party that is not involved in the conduction of the study. Training for CAPD exchange will be performed according to our routine clinical practice.

## Clinical follow up

Patients will be followed at 0, 4, 8, 16, 24, 32, 40 and 52 weeks. Except for the dialysis solution preparation, the clinical management will be identical for the two groups. During each follow up visit, we will measure body weight, blood pressure, presence of edema (semi-quantitative score from 0 to 3+), and compliance to dialysis exchange by direct questioning. Hemoglobin level, serum electrolytes, urea and creatinine will be checked upon each clinic visit.

*Measurement of Body Composition*

Body composition will be assessed at 4, 24 and 52 weeks. We will use the Body Composition Monitor (Fresenius Medical Care, Germany). Briefly, electrodes are attached to one hand and one foot with the patient in a supine position. After patient cable is connected, the measurement would complete automatically in 2 minutes. We will compute the following parameters from this test:

- total body water (TBW), intracellular water (ICW), and extracellular water (ECW)
- lean tissue mass, adipose tissue mass, and volume of over hydration

*Measurement of VPW and CTR*

In our unit, all PD patients have yearly chest X-ray for the screening of tuberculosis. We will review the chest X-ray before PD and one year later. All radiographic examinations are performed with computed radiography equipment (Mobilett Plus, Siemens Medical Solutions Malvern, PA) using a standardized technique (75 kV, 4 mAs, 180-cm film-focus distance; broad tube focus). The images are assessed using a PACS (Magicview, model VA22E, Siemens Medical Solutions) viewer (2K monitor). Briefly, the right border of VPW is the point that superior vena cava crossed the right main bronchus. The left border is the point of subclavian artery exiting from aorta. The VPW is defined as the horizontal distance measured between the two points. The distance of CTR is determined by Danzer method [15].

*Pulse Wave Velocity Study*

Pulse wave velocity (PWV), an index of aortic stiffness, is measured at 4 and 52 weeks using an automatic computerized recorder and the results are analyzed using the Complior® SP program (Artech Medical, France). The method of PWV measurement has been described previously [16]. Briefly, pressure-sensitive transducers are placed over the neck (carotid artery), wrist (radial artery) and groin (femoral artery) with the patient in the supine position within one week of PET. PWV of the carotid-femoral and carotid-radial territory is calculated by dividing the distance between the sensors by the time corresponding to the period separating the start of the rising phase of the carotid pulse wave and that of the femoral and also the radial pulse waves. The test will be performed by the same observer.

## Assessment of peritoneal transport

Peritoneal transport will be assessed at 4 and 52 weeks. We will use the standard PET as described by Twardowski [17]. Dialysate-to-plasma ratios of creatinine (D/P) at 4 hours will be calculated after correction of glucose interference [18]. Mass transfer area coefficients of creatinine (MTAC) normalized for body surface area (BSA) will be calculated by the formula described by Krediet [19].

## Dialysis adequacy and nutritional status

Dialysis adequacy will be assessed at 4, 24 and 52 weeks by 24-hour dialysate and urine collections will be performed. Total Kt/V and weekly creatinine clearance (CCr) will be determined by standard methods. Residual glomerular filtration rate (GFR) will be calculated as the average of 24-hour urinary urea and creatinine clearance [20]. From the same 24-hour dialysate and urine collection, we will also compute the fat-free edema-free body mass (FEBM) and normalized protein nitrogen appearance (NPNA). FEBM will be measured by creatinine kinetics according to the formula of Forbes and Brunining [21]. NPNA will be determined by the Bergstrom’s formula [22].

In addition to FEBM and NPNA, nutritional status will also be represented by Subjective global assessment (SGA) score, the comprehensive malnutrition-inflammation score (MIS), serum albumin and C-reactive protein (CRP) at 4, 24 and 52 weeks. The 4-item 7-point SGA scoring system, which is validated in CAPD patients [23], will be used. The calculation of MIS has been described previously [24]. Briefly, MIS consists of 4 main parts and 10 components, all will be measured by the Tina-quant CRP (Latex) ultra-sensitive assay (Roche Diagnostics GmbH, Mannheim, Germany)

*Clinical Outcome*

The primary outcome measures are the change in body composition and arterial pulse wave velocity. Secondary outcomes include nutritional and adequacy indices, peritoneal transport characteristics, residual renal function, peritonitis-free survival, hospitalization, and actuarial and technique survival. Technique failure is defined as transfer to long-term hemodialysis.

## Statistics

Statistical analysis will be performed by SPSS for Windows software version 15.0 (SPSS Inc., Chicago, IL). All data will be expressed in mean ± standard deviation unless otherwise specified. Parameters between groups are compared by Chi-square test, Student’s t test, or Mann-Whitney U test as appropriate.

*Justification of sample size*

The sample size is estimated by the Power Analysis and Sample Size for Windows software (PASS 2000, NCSS, Kaysville, Utah). Based on our previous study on hemodialysis [25], aortic PWV is expected to be 10.2 ± 1.6 m/sec. We assume a difference of 1 m/sec in the PWV to be clinically meaningful. Group sizes of 45 achieve 80% power to detect such a difference of PWV, with a significance level (alpha) of 0.05. Allowing for 10% drop out rate, the study will require 100 patients in total.

TIMETABLE OF WORK

It takes 36 months to complete the study:

Enrollment of case October 2010 to June 2013

Follow up of case July 2013 to June 2014

Analysis and write up report July 2014 to September 2014

SUMMARY OF PROPOSED WORK

| Week | 0 | 4 | 8 | 16 | 24 | 32 | 40 | 52 |
| --- | --- | --- | --- | --- | --- | --- | --- | --- |
| CAPD training | **✓** |  |  |  |  |  |  |  |
| Clinical follow up, routine blood tests | **✓** | **✓** | **✓** | **✓** | **✓** | **✓** | **✓** | **✓** |
| 24-hour urine and dialysate collection |  | **✓** |  |  | **✓** |  |  | **✓** |
| Nutritional Assessment |  | **✓** |  |  | **✓** |  |  | **✓** |
| Body composition monitor |  | **✓** |  |  | **✓** |  |  | **✓** |
| Radiographic measurements |  | **✓** |  |  |  |  |  | **✓** |
| Arterial pulse wave velocity |  | **✓** |  |  |  |  |  | **✓** |
| Peritoneal equilibration test |  | **✓** |  |  |  |  |  | **✓** |

REFERENCE

1. Davies SJ, Bryan J, Phillips L, Russell GI. Longitudinal changes in peritoneal kinetics: the effects of peritoneal dialysis and peritonitis. Nephrol Dial Transplant 1996; 11: 498-506.
2. Breborowicz A, Oreopoulos DG. Biocompatibility of peritoneal dialysis solutions. Am J Kidney Dis 1996; 27: 738-743.
3. Liberek T, Topley N, Jorres A, Petersen MM, Coles GA, Gahl GM, Williams JD. Peritoneal dialysis fluid inhibition of polymorphonuclear leucocyte respiratory burst activation is related to the lowering of intracellular pH. Nephrol 1993; 65: 260-265.
4. Cappelli G, Bandiani G, Cancarini GC, Feriani M, Dell'Aquila R, Saffioti S, Spisni C, Stingone A, Orazi E, Ballocchi S, Renaux JL. Low concentrations of glucose degradation products in peritoneal dialysis fluids and their impact on biocompatibility parameters: prospective cross-over study with a three-compartment bag. Adv Perit Dial 1999; 15: 238-242.
5. van Biesen W, Kirchgessner J, Schilling H, Lage C, Lambert MC, Passlick-Deetjen J. Stay-Safe®, a new PCV-free system for PD: results of the multicenter trial. Perit Dial Int 1999; 19(Suppl 1): S43.
6. Lage C, Pischetsrieder M, Aufricht C, Jorres A, Schilling H, Passlick-Deetjen J. First in vitro and in vivo experiences with Stay-Safe Balance, a pH-neutral solution in a dual-chambered bag. Perit Dial Int 2000; 20 (Suppl 5): S28-32.
7. Alscher DM, Pauli-Magnus C, Kirchgessner J, Kuhlmann U, Mettang T. A new lactate-based, plasticizer-free, neutral peritoneal dialysis fluid provided in a two-compartment system: effect on peripheral leukocyte function. Nephron 2000; 86: 62-69.
8. Passlick-Deetjen J, Pischetsrieder M, Witowski J, Bender TO, Jorres A, Lage C. In vitro superiority of dual-chambered peritoneal dialysis solution with possible clinical benefits. Perit Dial Int. 2001; 21 (Suppl 3): S96-101.
9. Wieczorowska-Tobis K, Polubinska A, Schaub TP, Schilling H, Wisniewska J, Witowski J, Passlick-Deetjen J, Breborowicz A. Influence of neutral-pH dialysis solutions on the peritoneal membrane: a long-term investigation in rats. Perit Dial Int 2001; 21 (Suppl 3): S108-113.
10. Mortier S, De Vriese AS, Van de Voorde J, Schaub TP, Passlick-Deetjen J, Lameire NH. Hemodynamic effects of peritoneal dialysis solutions on the rat peritoneal membrane: role of acidity, buffer choice, glucose concentration, and glucose degradation products. J Am Soc Nephrol 2002; 13: 480-489.
11. Schmitt CP, Haraldsson B, Doetschmann R, Zimmering M, Greiner C, Boswald M, Klaus G, Passlick-Deetjen J, Schaefer F. Effects of pH-neutral, bicarbonate-buffered dialysis fluid on peritoneal transport kinetics in children. Kidney Int 2002; 61: 1527-1536.
12. Williams JD, Topley N, Craig KJ, Mackenzie RK, Pischetsrieder M, Lage C, Passlick-Deetjen J. The Euro-Balance Trial: the effect of a new biocompatible peritoneal dialysis fluid (balance) on the peritoneal membrane. Kidney Int 2004; 66: 408-418.
13. Szeto CC, Chow KM, Lam CW, Leung CB, Kwan BC, Chung KY, Law MC, Li PK. Clinical biocompatibility of a neutral peritoneal dialysis solution with minimal glucose-degradation-products – a one-year randomized control trial. Nephrol Dial Transplant 2007; 22: 552-559.
14. Ates K, Nergizoglu G, Keven K, Sen A, Kutlay S, Erturk S, Duman N, Karatan O, Ertug AE. Effect of fluid and sodium removal on mortality in peritoneal dialysis patients. Kidney Int 2001;60: 767-776.
15. Philbin EF, Garg R, Danisa K, Denny U, Gosselin G, Hassapoyannes C, Homey A, Johnstone DE, Lang RM, Ramanathan K, Safford RE, Sarma RJ, Weiss R, Williford WO, Fleg JL. The relationship between cardiothoracic ratio and left ventricular ejection fraction in congestive heart failure. Arch Intern Med 1998; 158: 501-506.
16. Covic A, Gusbeth-Tatomir P, Goldsmith DJ. Arterial stiffness in renal patients: an update. Am J Kidney Dis 2005; 45: 965-977.
17. Twardowski ZJ, Nolph KD, Prowant B, Ryan L, Moore H, Nielsen MP: Peritoneal equilibration test. Perit Dial Bull 7: 138-147, 1987.
18. Mak TW, Cheung CK, Cheung CM, Leung CB, Lam CW, Lai KN: Interference of creatinine measurement in CAPD fluid was dependent on glucose and creatinine concentrations. Nephrol Dial Transplant 12: 184-186, 1997.
19. Krediet RT, Boeschoten EW, Zuyderhoudt FMJ, Strackee J, Arisz L. Simple assessment of the efficacy of peritoneal transport in continuous ambulatory peritoneal dialysis patients. Blood Purification 1986; 4: 194-203.
20. Van Olden RW, Krediet RT, Struijk DG, Arisz L. Measurement of residual rneal function in patients treated with continuous peritoneal dialysis. J Am Soc Nephrol 1996; 7: 745-748.
21. Forbes GB, Brunining GJ. Urinary creatinine excretion and lean body mass. Am J Clin Nutr 1976; 29: 1359-1366.
22. Bergstrom J, Heimburger O, Lindholm B. Calculation of the protein equivalent of total nitrogen appearance from urea appearance. Which formulas should be used? Perit Dial Int 1998; 18: 467-473.
23. Enia G, Sicus C, Alati G, Zoccali C. Subjective global assessment of nutrition in dialysis patients. Nephrol Dial Transplant 1993; 8: 1094-1098.
24. Kalantar-Zadeh K, Kopple JD, Block G, Humphreys MH. A malnutrition-inflammation score is correlated with morbidity and mortality in maintenance hemodialysis patients. Am J Kidney Dis 2001; 38: 1251-1263.
25. Li PKT, Cheng YL, Leung CB, Szeto CC, Chow KM, Kwan BC, Ng ES, Fok QW, Poon YL, Yu AW. Effect of membrane permeability on inflammation and arterial stiffness: a randomized trial. Clin J Am Soc Nephrol 2010; 5: 652-658.
