# Supplementary material for: The Effect of Neutral Peritoneal Dialysis Solution with Low Glucose-Degradation-Product on the Fluid Status and Body Composition – A Randomized Control Trial
Source: PLoS One. 2015 Oct 28;10(10):e0141425. doi: 10.1371/journal.pone.0141425 (PMC4625015; doi:10.1371/journal.pone.0141425)
Supplement: S1 Table — (DOCX) [file pone.0141425.s005.docx]

**S1 Table. Biochemical composition of the peritoneal dialysis solutions.**

|  | StaySafe® | Balance® |
| --- | --- | --- |
| Na^+^ (mmol/l) | 134 | 134 |
| Ca^2+^ (mmol/l) | 1.25 | 1.25 |
| Mg^2+^ (mmol/l) | 0.5 | 0.5 |
| Cl^-^ (mmol/l) | 100.5 | 100.5 |
| lactate (mmol/l) | 35 | 35 |
| pH | 5.5 | 7.0 |
